# Norisoboldine Suppresses Osteoclast Differentiation through Preventing the Accumulation of TRAF6-TAK1 Complexes and Activation of MAPKs/NF-κB/c-Fos/NFATc1 Pathways

**DOI:** 10.1371/journal.pone.0059171

**Published:** 2013-03-11

**Authors:** Zhi-feng Wei, Bei Tong, Yu-feng Xia, Qian Lu, Gui-xin Chou, Zheng-tao Wang, Yue Dai

**Affiliations:** 1 State Key Laboratory of Natural Medicines, Department of Pharmacology of Chinese Materia Medica, China Pharmaceutical University, Nanjing, China; 2 Institute of Chinese Materia Medica, Shanghai University of Traditional Chinese Medicine, Shanghai, China; University of Leuven, Rega Institute, Belgium

## Abstract

Norisoboldine (NOR) is the main alkaloid constituent in the dry root of *Lindera aggregata* (Sims) Kosterm. (*L. strychnifolia* Vill.). As reported previously, orally administered NOR displayed a robust inhibition of joint bone destruction present in both mouse collagen-induced arthritis and rat adjuvant-induced arthritis with lower efficacious doses than that required for ameliorating systemic inflammation. This attracted us to assess the effects of NOR on differentiation and function of osteoclasts, primary effector cells for inflammatory bone destruction, to get insight into its anti-rheumatoid arthritis mechanisms.

Both RAW264.7 cells and mouse bone marrow-derived macrophages (BMMs) were stimulated with RANKL (100 ng/mL) to establish osteoclast differentiation models. ELISA, RT-PCR, gelatin zymography, western blotting, immunoprecipitation and EMSA were used to reveal related signalling pathways. NOR (10 and 30 µM), without significant cytotoxicity, showed significant reduction of the number of osteoclasts and the resorption pit areas, and it targeted osteoclast differentiation at the early stage. In conjunction with the anti-resorption effect of NOR, mRNA levels of cathepsin K and MMP-9 were decreased, and the activity of MMP-9 was attenuated. Furthermore, our mechanistic studies indicated that NOR obviously suppressed the ubiquitination of TRAF6, the accumulation of TRAF6-TAK1 complexes and the activation of ERK and p38 MAPK, and reduced the nuclear translocation of NF-κB-p65 and DNA-binding activity of NF-κB. However, NOR had little effect on expressions of TRAF6 or the phosphorylation and degradation of IκBα. Moreover, NOR markedly inhibited expressions of transcription factor NFATc1, but not c-Fos. Intriguingly, the subsequent nuclear translocations of c-Fos and NFATc1 were substantially down-regulated. Hence, we demonstrated for the first time that preventing the differentiation and function of osteoclasts at the early stage was an important anti-bone destruction mechanism of NOR, which might be attributed to inhibition of ubiquitination of TRAF6, the accumulation of TRAF6-TAK1 complexes and the activation of MAPKs/NF-κB/c-Fos/NFATc1 pathways.

## Introduction

Severe erosion of bone and articular cartilage is the pathological hallmark of rheumatoid arthritis (RA), and ultimately leads to reduced mobility of affected patients [1], [2]. Increasing evidence indicates that osteoclasts play a crucial role in local bone erosion. In RA, abundant osteoclasts are found within the synovial tissues at sites adjacent to bone, creating resorption pits and local bone erosion followed by degradation of the bone matrix and calcium solubilization. In contrast, evident bone erosion did not appear in osteoclast-deficient mice with a serum transfer model of arthritis [3]–[7]. Therefore, osteoclasts are considered to be a type of pivotal target cells for anti-RA remedies.

Receptor activator of nuclear factor-kB (NF-κB) ligand (RANKL) has well been identified as a major inducer for osteoclast differentiation. Many other cytokines and factors, such as macrophage colony-stimulating factor (M-CSF), tumor necrosis factor alpha (TNF-α), interleukin-1 (IL-1) and parathyroid hormone-related peptide, work as additional enhancers [8]–[11]. Binding of RANKL with its receptor RANK induces the activation and accumulation of adaptor proteins TRAF6-TAK1 complexes, leading to the activation of NF-κB and MAPK pathways and the subsequent up-regulation of transcription factors c-Fos and NFATc1 during osteoclastogenesis. The activation of above-mentioned signalling pathways directly regulates a number of osteoclastogenesis-related marker genes, including TRAP, MMP-9 and cathepsin K, which lead to the formation of bone resorption pits during osteoclast differentiation. Denosumab, a fully human monoclonal antibody with a high affinity and specificity for RANKL, has been demonstrated to prevent structural damage in patients suffering from RA when added to ongoing methotrexate treatment [12], [13]. Targeted modulation of various signalling pathways to regulate osteoclastogenesis-related gene expressions may be helpful for the treatment of bone erosion in RA.

Radix Linderae is the dry root of *Lindera aggregata* and frequently used in traditional Chinese medicine. It contains a series of components, including alkaloids, volatile oils and sesquiterpene esters [14], [15]. Norisoboldine (NOR), as the major isoquinoline alkaloid in Radix Linderae, has a proven ability to ameliorate bone destruction in mouse collagen-induced arthritis (CIA) and rat adjuvant-induced arthritis (AIA) at lower doses than that required for repressing systemic inflammation, and to suppress the production of pro-inflammatory cytokine IL-6 in fibroblast-like synoviocytes from AIA rats through PKC/MAPK/NF-κB-p65/CREB pathways [16], [17]. The present study was performed to explore effects and precise mechanism of NOR on osteoclast differentiation and elucidate the reasons for its anti-bone destruction mechanisms in RA.

## Results

### Effects of NOR on tartrate resistant acid phosphatase (TRAP) activity in RAW264.7 cells induced by RANKL

**Figure 1:**
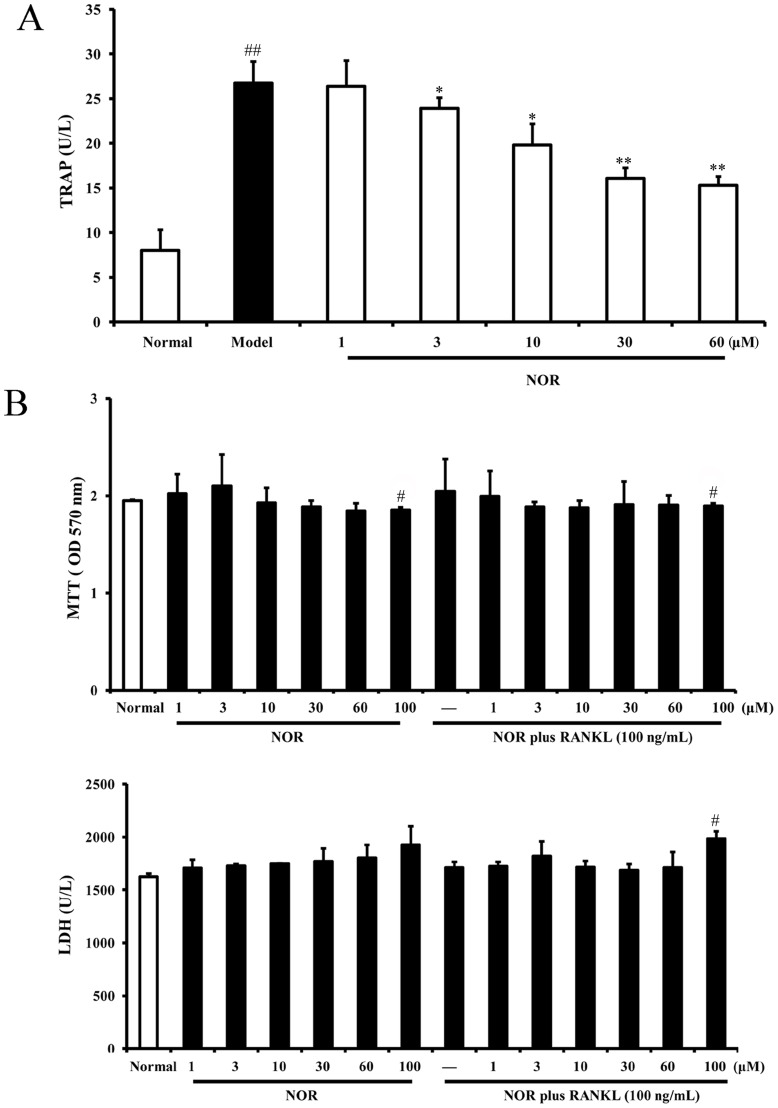
Effects of norisoboldine (NOR) on TRAP activity in RAW264.7 cells induced by RANKL. (**A**) RAW264.7 cells were treated with NOR (1, 3, 10, 30 and 60 µM) in the presence or absence of RANKL (100 ng/mL) for 24 h. The cells were lysed, and TRAP activity was detected using kits according to the manufacturer's instructions. (**B**) RAW264.7 cells were treated with NOR (1, 3, 10, 30, 60 and 100 µM) in the presence or absence of RANKL (100 ng/mL) for 72 h. Cytotoxicity was determined by MTT and LDH assays. Data were expressed as means±S.D. of three independent experiments. *^#^p*<0.05, *^##^p*<0.01 *vs*. normal; ^*^p<0.05, ^**^p<0.01 *vs.* model.

Tartrate resistant acid phosphatase (TRAP) is a well-known enzyme that widely acccepted to be a histochemical marker of osteoclasts, and it can be significantly elevated by RANKL stimulation. Initially, we examined the effects of NOR (3, 10, 30 and 60 µM) on TRAP activity and determined the concentrations required for inhibiting osteoclast differentiation. As depicted in Fig. 1A, RANKL (100 ng/mL) stimulation resulted in a dramatic enhancement of TRAP activity in RAW264.7 cells. NOR (3, 10, 30 and 60 µM) treatments obviously down-regulated TRAP activity in a concentration-dependent manner by 16.4%, 34.6%, 74.9% and 77.1%, respectively. Of note, at concentrations of 30 and 60 µM, it showed near inhibitory percentages.

To exclude the possibility that inhibition of NOR on TRAP activity was due to cytotoxicity, the viability of RAW264.7 cells in the presence or absence of RANKL was tested using both MTT and LDH assays. The results indicated that NOR-induced cytotoxicity was negligible below the concentration of 100 µM (Fig. 1B). Therefore, in the subsequent experiments, NOR (3, 10 and 30 µM) were used.

### Effects of NOR on osteoclastogenesis of RAW264.7 cells and mouse bone marrow-derived macrophages (BMMs) induced by RANKL

**Figure 2:**
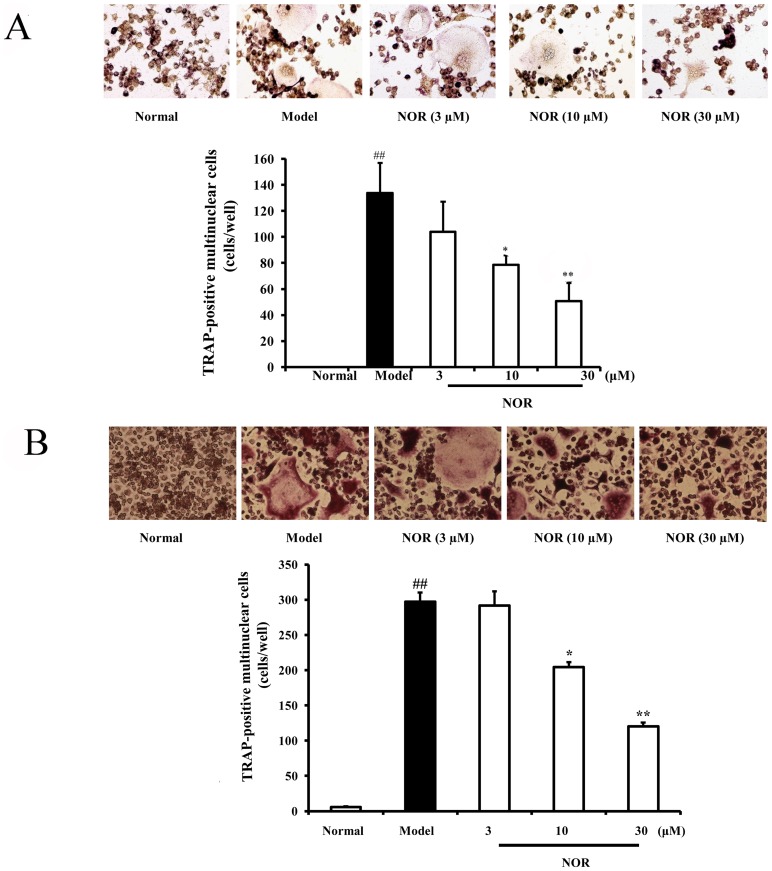
Effects of norisoboldine (NOR) on osteoclastogenesis of RAW264.7 cells and bone marrow-derived macrophages (BMMs) induced by RANKL. (**A**) RAW264.7 cells were treated with NOR (3, 10 and 30 µM) in the presence or absence of RANKL (100 ng/mL) for 5 days. (**B**) BMMs were treated with NOR (3, 10 and 30 µM) in the presence or absence of RANKL (100 ng/mL) for 5 days. Osteoclasts were identified using TRAP staining according to the manufacturer's instructions. TRAP-positive multinucleated cells with greater than three nuclei were counted as osteoclasts. Data were expressed as means±S.D. of three independent experiments. *^##^p*<0.01 *vs*. normal; ^*^p<0.05, ^**^p<0.01 *vs.* model.

Data in mountain have shown that osteoclasts are derived from monocyte-macrophage lineage cells. Accordingly, in our study, both kinds of monocyte-macrophage lineage cells RAW264.7 cells and BMMs were used to establish models of osteoclast differentiation. As shown in Fig. 2A & 2B, RANKL (100 ng/mL) stimulation for 5 days led to obvious formation of TRAP-positive multinuclear osteoclasts from either RAW264.7 cells or BMMs. NOR (10 and 30 µM) treatments produced a marked suppression of the number of osteoclasts derived from either RAW264.7 cells or BMMs. The inhibitory percentages of NOR (10 and 30 µM) were 41.3%, 62.4% and 31.3%, 59.6%, respectively.

### Effects of NOR on stages of osteoclastogenesis of RAW264.7 cells and BMMs induced by RANKL

**Figure 3:**
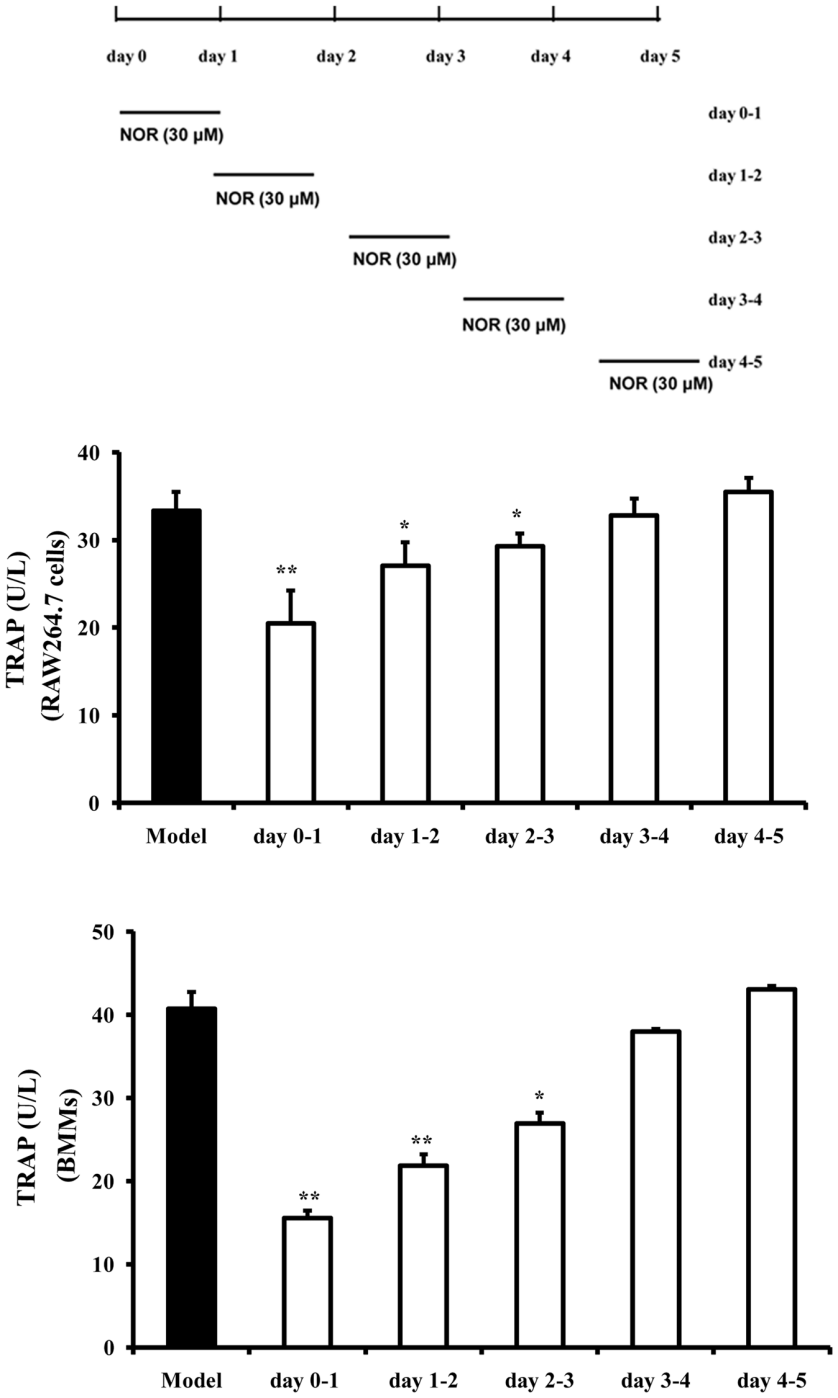
Effects of norisoboldine (NOR) on stages of osteoclastogenesis of RAW264.7 cells and bone marrow-derived macrophages (BMMs) induced by RANKL. RAW264.7 cells and BMMs were seeded into 24-well plates, and NOR (30 µM) was added into the cultures that treated with RANKL (100 ng/mL) at 5 different time points. After exposure to NOR for 24 h, the culture media containing NOR was washed out and changed to NOR free culture media.The cells were continued to be stimulated with RANKL (100 ng/mL). Untill day 5, cells were lysed in 0.2% Triton X-100, and TRAP activity was detected using kits according to the manufacturer's instructions. Data were expressed as means±S.D. of three independent experiments. ^*^p<0.05, ^**^p<0.01 *vs.* model.

To further clarify the stage of osteoclastogenesis that NOR preferentially affected, we added NOR (30 µM) into RANKL (100 ng/mL)-treated RAW264.7 cells and BMMs cultures at 5 different time points. After exposure to NOR for 24 h, the culture media containing NOR was washed out and changed to NOR free culture media. Our results revealed that NOR (30 µM) was able to inhibit TRAP activity when the cells were exposed at day 0–1, day 1–2 and day 2–3. The inhibitory percentages in RAW264.7 cells and BMMs were 38.7%, 18.9%, 12.3% and 61.9%, 46.3%, 33.8% at day 0–1, day 1–2 and day 2–3, respectively (Fig. 3). These findings indicated that NOR notly suppresses RANKL-induced osteoclastogenesis by targeting the early stage.

### Effects of NOR on the bone resorbing function of osteoclastogenesis of RAW264.7 cells induced by RANKL

**Figure 4:**
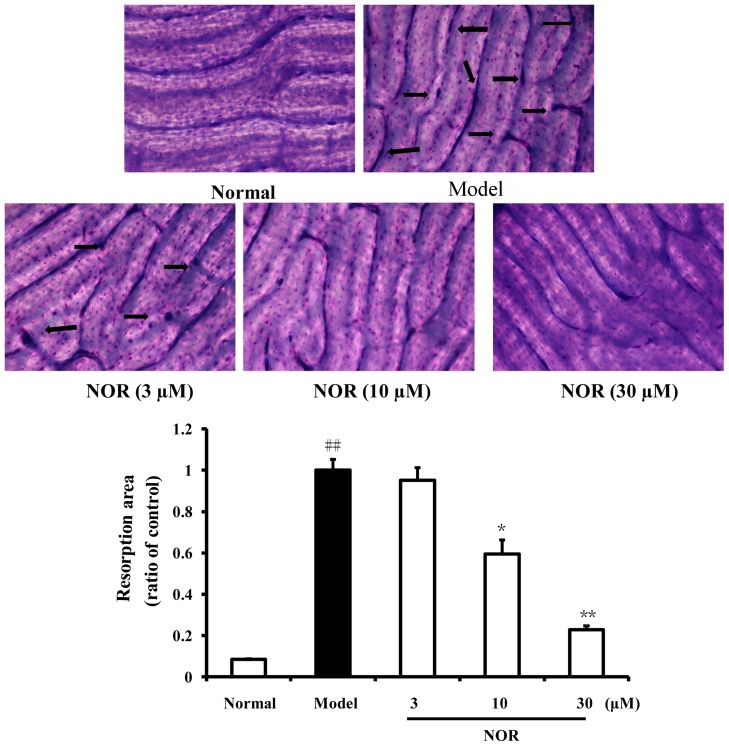
Effects of norisoboldine (NOR) on bone resorbing function of osteoclastogenesis of RAW264.7 cells induced by RANKL. RAW264.7 cells were seeded on bovine bone slices and treated with NOR (3, 10 and 30 µM) in the presence or absence of RANKL (100 ng/mL). After cultured for 5 days, the cells were removed from bone slices, and the slices were stained with toluidin blue. The areas were calculated by Image-Pro Plus 6.0. Data were expressed as means±S.D. of three independent experiments. *^##^p*<0.01 *vs.* normal; ^*^p<0.05, ^**^p<0.01 *vs.* model.

The formation of bone resorption pits occurs in conjunction with osteoclastogenesis. To determine whether the NOR treatments affect the bone resorption function of osteoclasts, bone slice assay was used. RAW264.7 cells were plated on bone slices and stimulated with RANKL for 5 days in the presence or absence of NOR (3, 10 and 30 µM). As Fig. 4 depicted, numerous resorption pits were formed on the bone slices. NOR (10 and 30 µM) treatments strongly diminished areas of bone resorption pits resulting from RANKL-induced RAW264.7 cells. The inhibitory percentages of NOR, at the concentrations of 10 and 30 µM, were 40.4% and 77.2%, respectively.

### Effects of NOR on the expressions of osteoclastogenesis-related genes in RAW264.7 cells induced by RANKL

**Figure 5:**
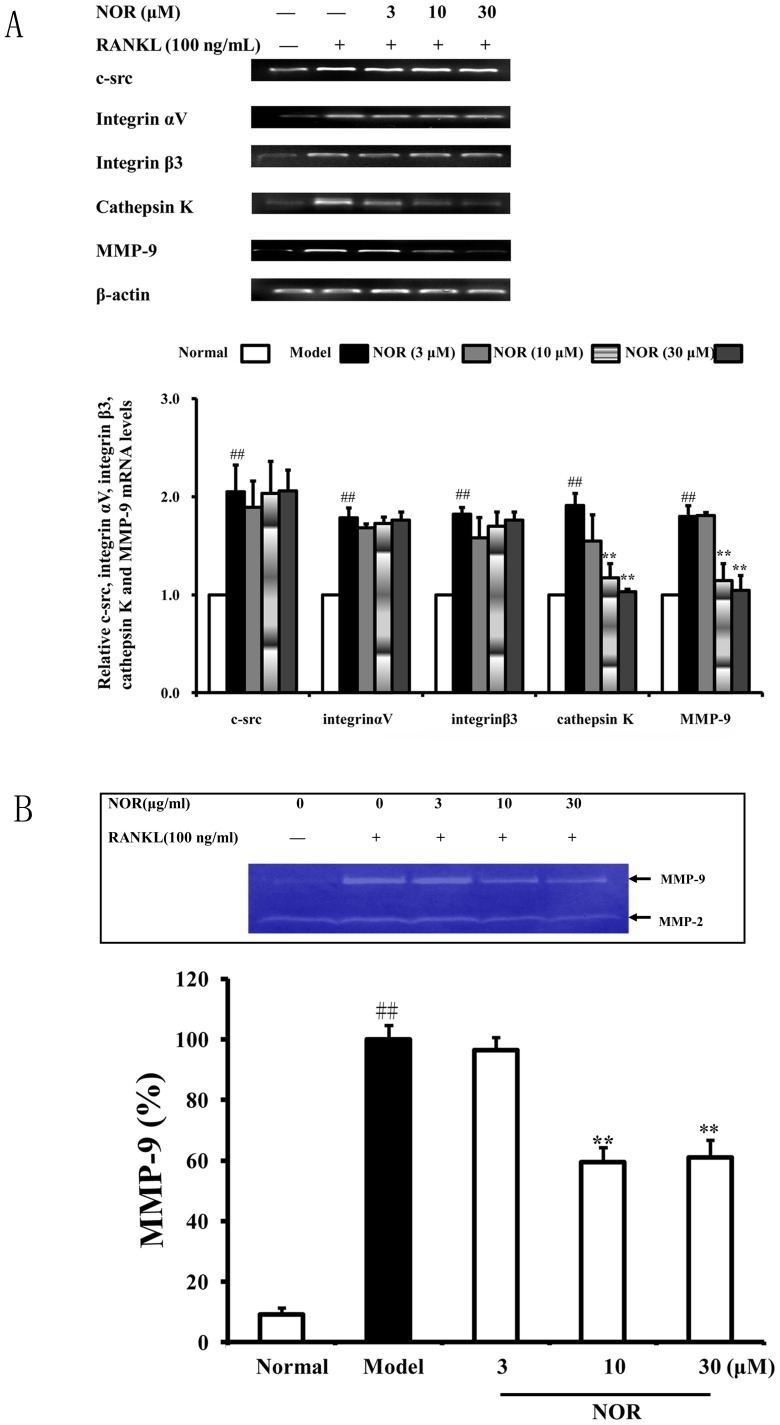
Effects of norisoboldine (NOR) on the expressions of osteoclastogenesis-related genes in RAW264.7 cells induced by RANKL. RAW264.7 cells were treated with NOR (3, 10 and 30 µM) in the presence or absence of RANKL (100 ng/mL) for 24 h. (**A**) The mRNA expressions of c-src, integrin αV, integrin β3, cathepsin K and MMP-9 were determined using RT-PCR assay. (**B**) The activity of secreted MMP-9 was detected using gelatin zymography assay. Data were expressed as means±S.D. of three independent experiments. ^##^ *p*<0.01 *vs.* normal; ^*^p<0.05, ^**^p<0.01 *vs.* model.

During osteoclastogenesis, the synthesis and secretion of adhesion molecules and matrix-degrading enzymes, such as c-src, integrin αVβ3, cathepsin K and MMP-9, are closely related to the eventual formation of bone resorption pits. Therefore, in the present study, we assessed the effects of NOR on the expressions of these molecules in RAW264.7 cells induced by RANKL. As shown in Fig. 5A, RANKL (100 ng/mL) stimulation led to significant up-regulation of the mRNA expressions of c-src, integrin αV, integrin β3, cathepsin K and MMP-9. Moreover, NOR (10 and 30 µM) treatments substantially reduced the mRNA levels of the matrix-degrading enzymes cathepsin K and MMP-9, but not that of adhesion molecules c-src, integrin αv and integrin β3. These results suggested that NOR suppressed the bone resorption function of osteoclasts by inhibiting the production of matrix-degrading enzymes, but not adhesion and migration of osteoclasts.

To ascertain whether NOR treatments affect the activity of MMP-9, the gelatin zymography assay was used. As shown in Fig. 5B, RANKL (100 ng/mL) stimulation led to a significant increase of MMP-9 activity in RAW264.7 cells, and NOR (10 and 30 µM) treatments markedly reduced MMP-9 activity. In contrast, the activity of MMP-2 was affected by neither the RANKL stimulation nor the NOR treatments.

### Effects of NOR on the expression and ubiquitination of TRAF6 as well as accumulation of TRAF6-TAK1 complexes in RAW264.7 cells induced by RANKL

**Figure 6:**
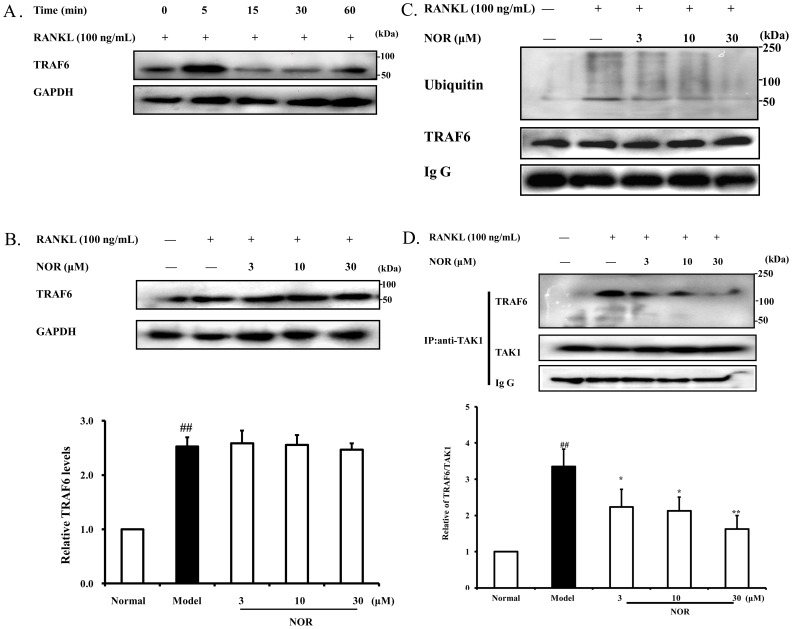
Effects of norisoboldine (NOR) on the expression and ubiquitination of TRAF6 as well as accumulation of TRAF6-TAK1 complexes in RAW264.7 cells induced by RANKL. (**A**) Time course of the expressions of TRAF6 in RANKL-induced RAW264.7 cells. Cells were pre-treated with RANKL (100 ng/mL), and cell lysates were collected at the time points indicated. Expressions of TRAF6 were detected by western blotting assay. (**B**) RAW264.7 cells were pre-treated with NOR (3, 10 and 30 µM) for 24 h and stimulated with RANKL (100 ng/mL) for 5 min. Expression of TRAF6 were detected by western blotting assay. (**C**) RAW264.7 cells were pre-treated with NOR (3, 10 and 30 µM) for 24 h and then stimulated with RANKL (100 ng/mL) for 5 min. Cells were lysed, and extracts were immunoprecipitated with antibody for TRAF6. The ubiquitination of TRAF6 was detected by immunoblotting with antibody. (**D**) RAW264.7 cells were pre-treated with NOR (3, 10 and 30 µM) for 24 h and then stimulated with RANKL (100 ng/mL) for 5 min. Cells were lysed and extracts were immunoprecipitated with antibody for TAK1. Co-precipitated TRAF6 was detected by immunoblotting with antibody. Data were expressed as means±S.D. of three independent experiments. ^##^ *p*<0.01 *vs.* normal; ^*^ *p*<0.05, ^**^p<0.01 *vs.* model.

RANKL acts by binding to its receptor RANK, resulting in increased expression and ubiquitination of TRAF6 and the accumulation of TRAF6-TAK1 (a MAPK kinase kinase) complexes, which activate the downstream signalling molecules such as PI3K, MAPKs and NF-κB. As shown in Fig. 6A, RANKL (100 ng/mL) stimulation obviously up-regulated the expression of TRAF6 in RAW264.7 cells, reaching peak accumulation at 5 min. In contrast, NOR (3, 10 and 30 µM) pre-treatments had little effect on the expression of TRAF6 (Fig. 6B), but the ubiquitination of TRAF6 and accumulation of TRAF6-TAK1 complexes were significantly suppressed by NOR. The inhibitory percentages of NOR (3, 10 and 30 µM) on the accumulation of TRAF6-TAK1 complexes were 33.4%, 36.7% and 51.6%, respectively (Fig. 6C & 6D).

### Effects of NOR on activation of MAPKs in RAW264.7 cells induced by RANKL

**Figure 7:**
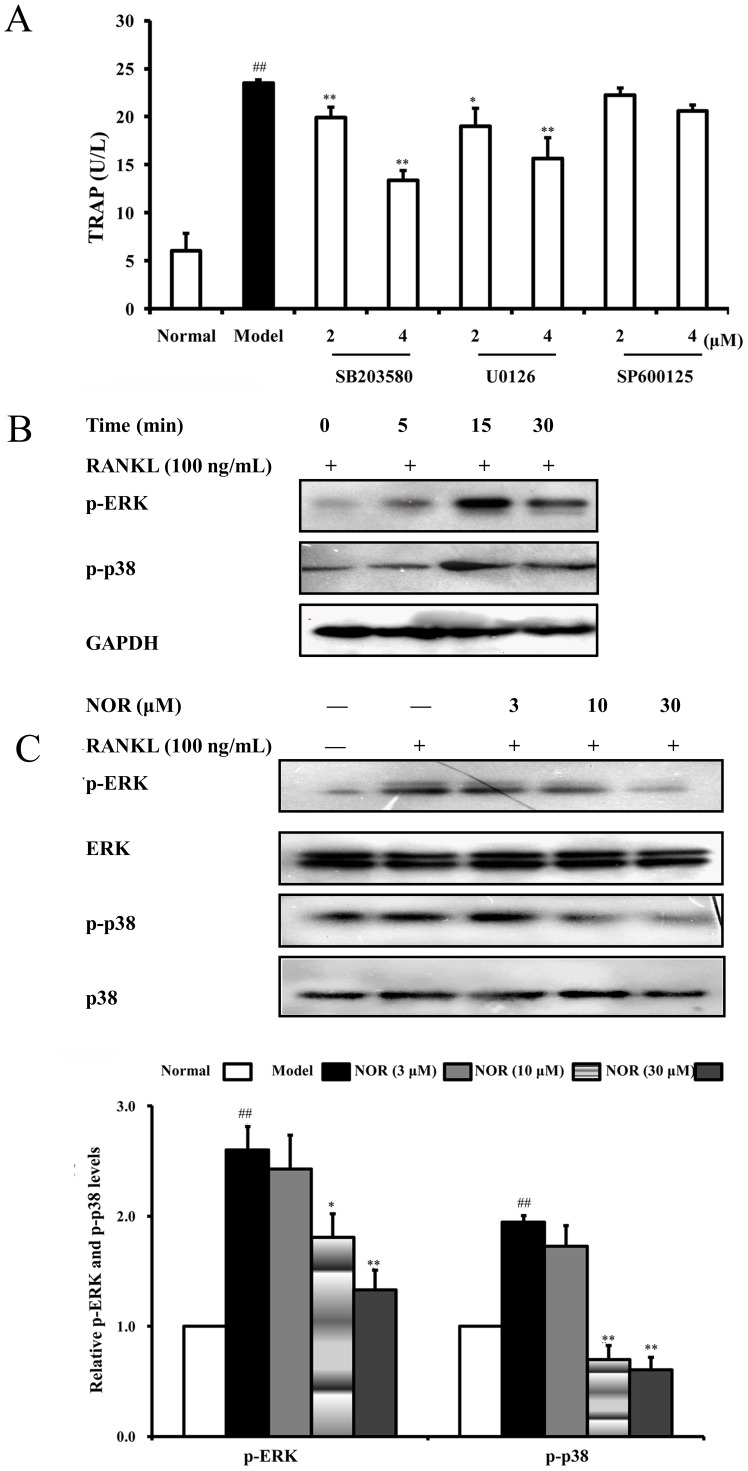
Effects of norisoboldine (NOR) on the activation of MAPKs in RAW264.7 cells induced by RANKL. (**A**) RAW264.7 cells were pre-treated with SB203580 (2 and 4 µM), U0126 (2 and 4 µM) and SP600125 (2 and 4 µM) in the presence or absence of RANKL (100 ng/mL) for 24 h. Cells were lysated, and TRAP activity was detected using kits according to the manufacturer's instructions. (**B**) Time course of the phosphorylations of p38 MAPK and ERK in RANKL-induced RAW264.7. Cells were treated with RANKL (100 ng/mL), and cell lysates were collected at the time points indicated. Phosphorylations of p38 MAPK and ERK were detected by western blotting assay. (**C**) RAW264.7 cells were pre-treated with NOR (3, 10 and 30 µM) for 24 h and stimulated with RANKL (100 ng/mL) for 15 min. Then, p38 MAPK, p-p38 MAPK, ERK and p-ERK were analyzed using western blotting assay. Data were the means±S.D. of three independent experiments. *^##^p*<0.01 *vs*. normal; ^*^ *p*<0.05, ^**^p<0.01 *vs*. model.

MAPKs (mainly including ERK, JNK and p38 MAPK) are located at the downstream of the TRAF6 signalling complexes, and they play an important role in RANKL-induced osteoclastogenesis by triggering a cascade reaction and up-regulating expressions of essential transcription factors c-Fos, MITF, PU.1 and NFATc1. In RANKL-stimulated RAW264.7 cells, SB203580 (a specific inhibitor of p38) and U0126 (a specific inhibitor of ERK), but not SP600125 (a specific inhibitor of JNK), reduced TRAP activity in a concentration-dependent manner (Fig. 7A). Our data strongly indicated that p38 MAPK and ERK participate in the regulation of RANKL-induced osteoclast differentiation. We found that RANKL stimulation led to evident phosphorylations of both p38 MAPK and ERK in RAW264.7 cells, peaking at 15 min. Moreover, NOR (10 and 30 µM) pre-treatments for 24 h remarkably inhibited the phosphorylations. The inhibitory percentages against p-p38 MAPK and p-ERK were 64.4%, 69.0% and 30.4%, 48.8%, respectively (Fig. 7B & 7C).

### Effects of NOR on the activation of the NF-κB pathway in RAW264.7 cells induced by RANKL

**Figure 8:**
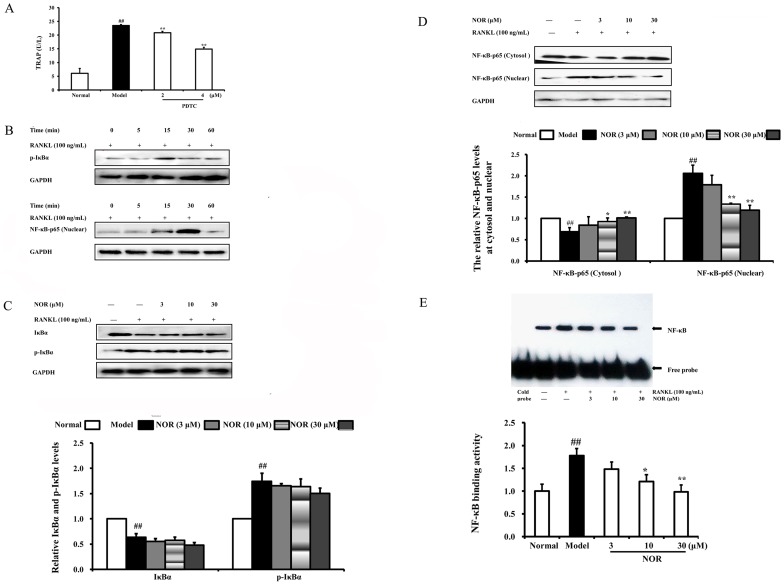
Effects of norisoboldine (NOR) on activation of NF-κB pathway in RAW264.7 cells induced by RANKL. (**A**) RAW264.7 cells were pre-treated with PDTC (2 and 4 µM) in the presence or absence of RANKL (100 ng/mL) for 24 h. Cells were lysated, and TRAP activity was detected using kits according to the manufacturer's instructions. (**B**) Time course of the phosphorylation of IκBα and expression of NF-κB-p65 (nuclear). RAW264.7 cells were treated with RANKL (100 ng/mL), and cell lysates were collected at the time points indicated. Phosphorylations of IκBα and the expression of NF-κB-p65 (nuclear) were detected by western blotting using specific antibodies. (**C**) RAW264.7 cells were pre-treated with NOR (3, 10 and 30 µM) for 24 h and stimulated with RANKL (100 ng/mL) for 15 min. IκBα and p-IκBα were analyzed by western blotting assay. GAPDH was used as the internal control. (**D**) RAW264.7 cells were pre-treated with NOR (3, 10 and 30 µM) for 24 h and then stimulated with RANKL (100 ng/mL) for 30 min. Expressions of NF-κB-p65 in cytosol and nuclear proteins were analyzed using western blotting assay. (**E**) RAW264.7 cells were pre-treated with NOR (3, 10 and 30 µM) for 24 h and stimulated with RANKL (100 ng/mL) for 30 min. The DNA binging activity of NF-κB in nuclear proteins was analyzed using EMSA. Data were expressed as means±S.D. of three independent experiments. ^##^ *p*<0.01 *vs*. normal; ^*^ *p*<0.05, ^**^p<0.01 *vs*. model.

In addition to MAPKs, NF-κB is also heavily involved in the regulation of osteoclastogenesis. The activation of NF-κB is linked to a sequential cascade that includes IKK-dependent IκBα phosphorylation, ubiquitination and proteolytic degradation, as well as the translocation of cytosolic NF-κB-p65 to nucleus. Our studies showed that PDTC (a specific inhibitor of NF-κB) significantly reduced the activity of TRAP, a histochemical marker of osteoclasts, in RANKL-induced RAW264.7 cells (Fig. 8A). Hence, the effects of NOR on the activation of NF-κB pathway were subsequently addressed. As shown in Fig. 8B, RANKL (100 ng/mL) stimulation resulted in a rapid and evident phosphorylation and degradation of IκBα, as well as the subsequent nuclear translocation of the NF-κB-p65 subunit in RAW264.7 cells. Intriguingly, NOR pre-treatments failed to affect RANKL-induced phosphorylation and degradation of IκBα (Fig. 8C). However, at concentrations of 10 and 30 µM, NOR treatment for 24 h markedly blocked NF-κB-p65 nuclear translocation in RAW264.7 cells, which was evidenced by the increased amounts of NF-κB-p65 in the cytoplasm and reduced amounts in the nucleus (Fig. 8D). Subsequently, DNA-binding activity of NF-κB was assessed using EMSA, and the results showed that RANKL stimulation strongly up-regulated the activity of NF-κB DNA-binding. This elevated activity of NF-κB DNA-binding was dramatically reduced by NOR (10 and 30 µM) pre-treatments (Fig. 8E). Taken together, these results indicated that the inhibition of the NF-κB pathway by NOR contributed to its suppression of osteoclastogenesis.

### Effects of NOR on the expressions of transcription factors c-Fos and NFATc1 in RAW264.7 cells induced by RANKL

**Figure 9:**
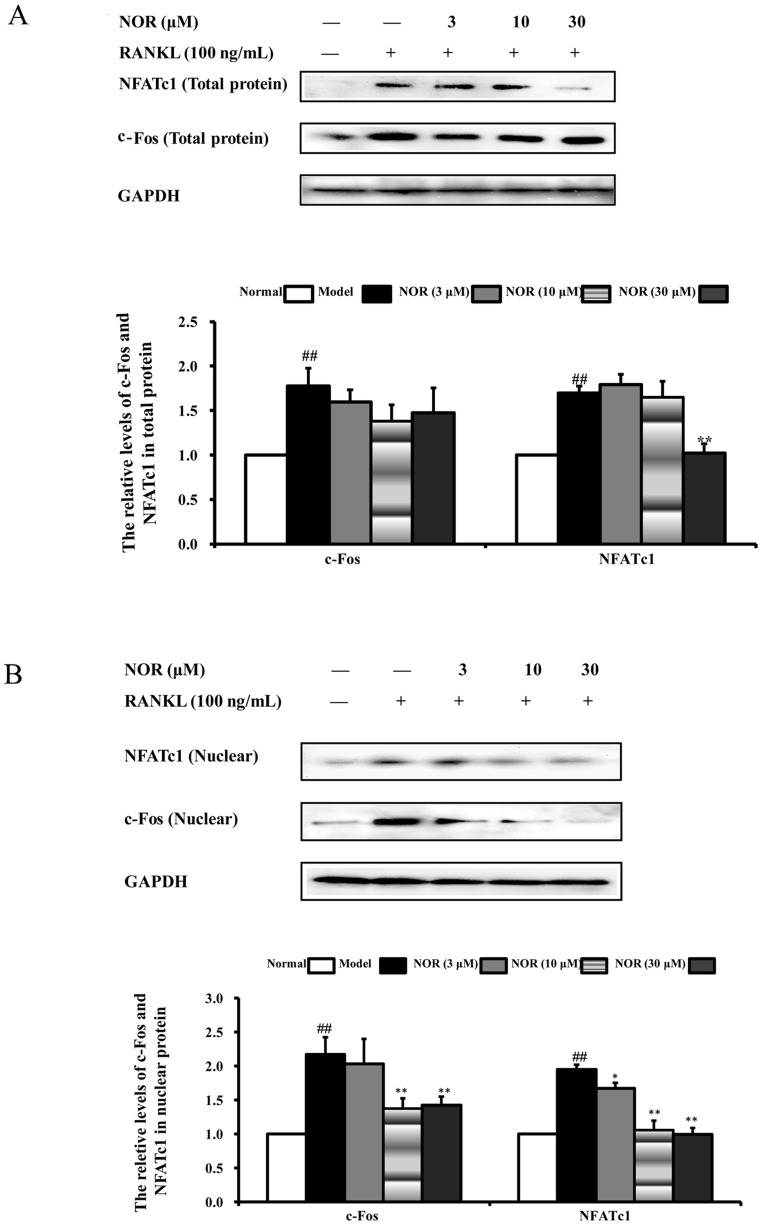
Effects of norisoboldine (NOR) on expressions of transcription factors c-Fos and NFATc1 in RAW264.7 cells induced by RANKL. RAW264.7 cells were treated with NOR (3, 10 and 30 µM) in the presence or absence of RANKL (100 ng/mL) for 24 h. Cytoplasm and nuclear proteins were extracted. Expressions of c-Fos and NFATc1 were analyzed using western blotting assay. GAPDH was used as the internal control. Data were expressed as means±S.D. of three independent experiments. ^##^ *p*<0.01 *vs*. normal; ^*^p<0.05, ^**^p<0.01 *vs*. model.

Binding of RANKL to RANK should activate several transcription factors that are responsible for promoting osteoclastic gene expression. However, they are not all activated within the same time phase: early response factors, such as c-Fos, are activated before late response factors, such as NFATc1. NFATc1 and c-Fos are identified as two of the most important osteoclasts-specific transcription factors [18]. In resting cells, both of them are restricted to the cytoplasm, and the activation of cells leads to their nuclear translocations. In our study, the expressions and nuclear translocations of these two transcription factors were assessed. As shown in Fig. 9A & 9B, RANKL stimulation for 24 h obviously up-regulated the expressions and nuclear translocations of both c-Fos and NFATc1 in RAW264.7 cells. Additionally, NOR (10 and 30 µM) treatments markedly inhibited the nuclear translocation of c-Fos by 36.9% and 31.3%, respectively, and NFATc1 by 46.1% and 49.2%, respectively. However, NOR treatments, at the highest concentration of 30 µM, only suppressed the expressions of NFATc1, but not that of c-Fos.

## Discussion

RA is a chronic autoimmune disease characterized by inflammation of the synovial tissues and destruction of the underlying cartilage and bone, that both resulting in functional impairment [1]–[3]. The goal of anti-rheumatic treatment is not only to attenuate the clinical symptoms of joint inflammation but also to inhibit the progression of joint destruction. Progressive destruction of cartilage and bone in RA results from elevated pro-inflammatory cytokines, synovial neovascularization, proteinase-mediated dissolution of articular cartilage matrix and osteoclast-mediated subchondral bone resorption. There is emerging interest for osteoclast as a key player in the erosive and inflammatory events leading to joint destruction in arthritis with special regard to RA. The accumulation of osteoclasts in rheumatoid synovial tissues and their activation associated with osteoclastogenic cytokines and chemokines at erosion sites suggest that they could be selected as curative therapeutic targets [19], [20]. The new drugs aiming to directly inhibit the differentiation and function of osteoclasts are fascinating. Radix Linderae has been used in China for the treatment of rheumatoid-related diseases for many years. NOR is the main active constituent of this herb medicine and has been previously demonstrated to inhibit bone destruction of RA at a lower efficacious dose than that required for ameliorating inflammation. Therefore, we examined the effects of NOR on the differentiation and function of osteoclasts and explored the underlying mechanisms.

Osteoclasts are unique multinuclear giant cells, which are derived from monocyte-macrophage lineage cells. Their differentiation from precursors can be induced by RANKL, a key factor that also controls the function and survival of mature osteoclasts. RANKL is highly expressed by fibroblast-like synoviocytes, osteoblasts and stromal cells in RA. It belongs to TNF super family and, as a result of proteolytic cleavage, is often detected as a soluble factor [21], [22]. As a ligand, RANKL interacts to its receptor RANK, a type I transmembrane receptor that presents on marrow monocytes and macrophages, and induces them to differentiate into osteoclasts [23]. Notably, until now, no other endogenous factors that can lead to osteoclast formation without RANKL participation have been found. Therefore, RANKL is used as a convincing stimulator in studies on differentiation and function of osteoclasts. In this study, both RAW264.7 cells and BMMs were adopted and induced by RANKL to establish models of osteoclast differentiation. Our results revealed that NOR (10 and 30 µM) treatments significantly reduced the number of TRAP-positive multinuclear osteoclasts, which mainly affected the early stage of osteoclast differentiation.

It is generally accepted that the formation of bone resorption pits occurs in conjunction with the process of osteoclast differentiation. The secretion of enzymes and the adhesion and migration of osteoclasts collectively result in bone resorption. Several proteolytic enzymes, including TRAP, cathepsin K, MMP-13 and MMP-9, have been demonstrated to play important roles in degrading the organic bone matrix. Among them, cathepsin K and MMP-9 have the highest levels in osteoclasts. Both cathepsin K and MMP-9 are known to be collagen-degrading enzymes, and they can directly degrade collagens in hard tissues that have been demineralized by v-ATPase during osteoclastic resorption. Cathepsin K knockout mice develop osteopetrosis because of the impaired matrix degradation; MMP-9 knockout mice show only transient disturbances in bone development [24]. In addition, the adhesion molecules integrin αV, integrin β3 and c-src play important roles in regulating bone resorption of osteoclasts by mediating their migration and adhesion. Integrin αV-, integrin β3- and c-src-deficient mice develop enhanced bone mass as a result of osteoclast dysfunction [25], [26]. In this study, RANKL stimulation in RAW264.7 cells led to the formation of many resorption pits on bone slices, and NOR (10 and 30 µM) treatments dramatically diminished areas of bone resorption pits. Results of RT-PCR and gelatin zymography assays further revealed that the anti-bone resorption effect of NOR was accompanied with not only decreased levels of matrix-degrading enzymes cathepsin K and MMP-9, but also attenuated activity of MMP-9. However, the levels of c-src, integrin αV and integrin β3 were not affected. We therefore postulated that the down-regulation of bone resorption activity of osteoclasts by NOR was associated with selective inhibition of matrix-degrading enzymes rather than the adhesion and migration of osteoclasts.

RANKL functions by binding to its receptor RANK. The intracellular domain of RANK includes two TRAF-binding domains, both of which specifically recognize different TRAF proteins [27]. The C-terminal region of RANK interacts with TRAF2 and TRAF5, while the TRAF6-binding domain resides in the middle of the RANK intracellular region. Overexpression of RANK C-terminal deletion mutants has shown that activation of the RANKL-mediated signalling pathway is associated with TRAF6. TRAF6-deficient mice exhibit severe osteopetrosis, and are defective in bone remodeling caused by impaired osteoclast function [28], [29]. On the other hand, TRAF6, as a unique member of a family of RING domain ubiquitin ligases, can catalyze polyubiquitin chains linked through Lys-63 of ubiquitin and then contribute to the activation of the signalling pathway at the downstream [30]. In addition, TGF-beta activated kinase 1 (TAK1), a member of the mitogen-activated protein kinase kinase kinase (MAPKKK) family, is recruited for the formation of TRAF6-TAK1 complexes after stimulated with RANKL in cells. The complexes will activate PI3K, MAPKs and NF-κB pathways. Based on our western blotting and immunoprecipitation data, RANKL stimulation indeed evoked rapid and significant expression and ubiquitination of TRAF6 and the accumulation of TRAF6-TAK1 complexes. NOR (3, 10 and 30 µM) pre-treatments had little effect on the expression of TRAF6, but significantly suppressed the ubiquitination of TRAF6 and subsequent accumulation of TRAF6-TAK1 complexes.

Three major subfamilies of MAPKs (p38 MAPK, ERK and JNK), located downstream of TRAF6 signalling, have been implicated as key regulators of various cellular responses, including cell proliferation, apoptosis, differentiation and migration [31]. These kinases also play pivotal roles in the development of osteoclasts and have been considered as key molecular targets for therapeutic application in inflammatory bone destruction [32]–[34]. Here, we found that SB203580 (a specific inhibitor of p38 MAPK) and U0126 (a specific inhibitor of ERK) but not SP600125 (a specific inhibitor of JNK), could significantly reduce TRAP activity in RANKL-induced RAW264.7 cells. These findings supported the notion that the activation of p38 MAPK and ERK plays a strong role in RANKL-induced osteoclastogenesis. Furthermore, NOR was shown to suppress the phosphorylations of p38 MAPK and ERK in RAW264.7 cells induced by RANKL.

As an important signalling mediator for inflammatory and immune responses, NF-κB is also thought to be one of the major transcription factors of RANKL-induced osteoclastogenesis. The activation of NF-κB refers to IκB (the inhibitor of NF-κB) phosphorylation, and the latter depends on the regulation of IKK (the kinase of IκB). IKK/IκB/NF-κB constitutes an organism system. RANKL stimulation results in IKK activation and causes IκBα phosphorylation. Then, the polyubiquitinated IκBα is degraded by proteasomes, and the dissociative NF-κB-p65 subunit enters into nucleus and binds to the particular position of DNA, and then start the target gene replication [35]. Mice lacking NF-κB-p65 globally show embryonic lethality, but radiation chimeras with p65-deficient bone marrow are viable. These p65^−/−^ chimeras have fewer osteoclasts and a significantly blunted osteoclastogenic response to RANKL injection [36]. The results of our studies showed that PDTC, a specific inhibitor of NF-κB, could significantly down-regulate TRAP activity in RANKL-induced RAW264.7 cells, strongly suggesting that the NF-κB pathway is involved in osteoclastogenesis. NOR pre-treatment failed to affect the phosphorylation and degradation of IκBα but markedly suppressed the following nuclear translocation of NF-κB-p65 and the DNA-binding activity of NF-κB as detected by western blotting and electrophoretic mobility shift assay (EMSA), respectively. These findings indicated that inhibiting the activation of NF-κB pathway contributed to the suppression of NOR against osteoclastogenesis.

Following the activation of the MAPK and NF-κB signalling pathways caused by RANKL, multiple osteoclastogenic transcription factors, such as c-Fos, NFATc1, MITF and PU.1, should be induced to mediate the formation of mature and functionally active osteoclasts [37]. Among them, c-Fos and NFATc1 play essential roles in osteoclast differentiation, and the absence of these components should arrest osteoclastogenesis. NFATc1, a member of the NFAT family, resides in endocardial cushion endothelial cells, T lymphocytes, osteoblasts, osteoclasts and others cells [38]. NFATc1-deficient embryonic stem cells fail to differentiate into osteoclasts, and ectopic expression of NFATc1 causes precursor cells to undergo efficient differentiation without RANKL stimulation [39]. c-Fos, a major component of the transcription factor AP-1, can induce the expressions of NFATc1 to regulate osteoclast differentiation after RANKL stimulation. Mice lacking c-Fos develop osteopetrosis as a result of a complete ablation of osteoclast formation [18]. Cells lacking c-Fos, but containing NFATc1, can differentiate into osteoclasts, indicating that NFATc1 is located downstream of c-Fos. Our results revealed that RANKL stimulation markedly up-regulated the expressions and nuclear translocations of both c-Fos and NFATc1, and NOR (30 µM) treatment only suppressed the expression of NFATc1 but not that of c-Fos. In contrast, NOR (10 and 30 µM) treatments dramatically suppressed the nuclear translocations of both c-Fos and NFATc1, as evidenced by their reduced amounts in the nucleus.

In conclusion, we confirmed that NOR was indeed able to suppress osteoclast differentiation by targeting at the early stage and inhibit osteoclastic-resorbing function via down-regulating the expressions of the bone matrix-degrading enzymes cathepsin K and MMP-9. The underlying mechanisms involved inhibiton of the ubiquitination of TRAF6, the accumulation of TRAF6-TAK1 complexes, and the activation of MAPKs/NF-κB/c-Fos/NFATc1 pathways.

## Materials and Methods

### Chemicals and reagents

**Figure 10:**
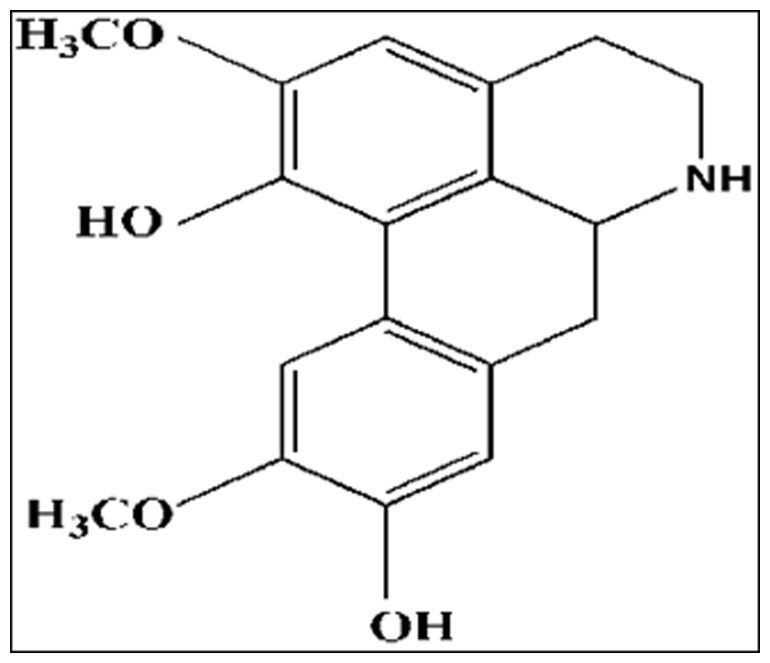
Chemical structure of norisoboldine.

NOR (purity>98%) was isolated and purified from Radix Linderae by the authors, and the structure was established by comparison of its spectral data (UV, IR, MS, 1H- and 13C-NMR) with previously reported data (Fig. 10) [40]; α-minimum essential medium (α-MEM), Dulbecco's Modified Eagle Media (DMEM) and fetal bovine serum (FBS) were purchased from Gibco BRL (Grand Island, USA); Mouse RANKL and M-CSF were purchased from PeproTech Int. (Connecticut, USA); Lactate dehydrogenase (LDH) kit was purchased from Nanjing Jiancheng Bioengineering Institute (Nanjing, China); Mouse TRAP ELISA was purchased from Dizhao Biotech CO., LTD., (Nanjing, China); 3-[4,5-dimetylthiazol-2-yl]-2,5-diphenyltetrazolium bromide (MTT) and Leukocyte acid phosphatase (TRAP) kit were purchased from Sigma Chemical Co. (St. Louis, MO, USA); Bovine bone slices were purchased from Nordic Biosciense (Herlev, Denmark); SB203580 (a specific inhibitor of p38 MAPK), U0126 (a specific inhibitor of ERK), and SP600125 (a specific inhibitor of JNK) were purchased from KangChen Bio-tech (Shanghai, China); PDTC (a specific inhibitor of NF-κB) was purchased from Enzo Life Sciences, Inc (Shanghai, China); ERK, JNK, p38, IκBα, NF-κB-p65, p-ERK, p-JNK, p-p38, p-IκBα and GAPDH monoclonal antibodies were purchased from KangChen Bio-tech (Shanghai, China); c-Fos, NFATc1 and TRAF6 monoclonal antibodies were purchased from Bioworld (Georgia, USA); TAK1 monoclonal antibody was purchased from Santa Cruz Biotechnology (CA, USA); Ubiquitin monoclonal antibody was purchased from abcam company (Cambridge, USA); Biotin 3′end DNA labeling kit and Lightshift EMSA optimization & control kit were purchased from Pierce (Rockford, USA); Nuclear and cytoplasmic protein extraction kit was purchased from Sangon Biotech (Shanghai, China); TRIzol reagent was purchased from Invitrogen (Carlsbad, CA); the M-MLV reverse transcriptase system and Taq polymerase were purchased from TransGen Biotech (Beijing, China); Enhanced chemiluminescent (ECL) plus reagent kits and peroxidase-conjugated secondary antibody were purchased from MultiSciences (Hangzhou, China). The other chemicals and reagents used were of analytical grade.

### Ethics statements

The animal experiments were conducted with the approval of the Animal Ethics Committee of Shanghai University of Traditional Chinese Medicine and conformed to the National Institute of Health guidelines on the ethical use of animals. All the animal experiments were made to minimize suffering and reduce the number of animals used.

### Animals

Male ICR mice, weighing 15–22 g, between the ages of 4 and 6 weeks, were purchased from the Comparative Medicine Centre of Yangzhou University. The mice were housed in a temperature- and humidity-controlled room and were allowed free access to a standard chow diet and water before the experiments.

### Cell cultures

The RAW264.7 cell line was obtained from American Type Culture Collection (ATCC, Manassas, VA, USA). The RAW264.7 Cells were cultured in DMEM supplemented with 10% FBS and maintained at 37°C in 5% CO_2_ humidified air.

Bone marrow cells were obtained from male ICR mice of 4- to 6-week-old which were sacrificed using ether anesthesia. The tibia and femur of mice were obtained and flushed with α-MEM containing 10% FBS. Cells were then centrifuged for 5 min at a speed of 1000 rpm. The precipitation was re-suspended with red blood cell lysis buffer and centrifuged for 5 min at a speed of 1000 rpm again. Precipitated cells were suspended in α-MEM supplemented with 10% FBS and cultured for 24 h. Non-adherent cells were collected and cultured for 3 days in the presence of M-CSF (50 ng/mL). Floating cells were discarded and adherent cells were classified as bone marrow-derived macrophages (BMMs).

In all the normal groups, the RAW264.7 cells were not treated, and the BMMs were treated with M-CSF (50 ng/mL); In all the model groups, the RAW264.7 cells were treated with RANKL (100 ng/mL), and the BMMs were treated with M-CSF (50 ng/mL) and RANKL (100 ng/mL).

### Cytotoxicity assay

The cytotoxic effect of NOR was evaluated using MTT and LDH assays [41]. RAW264.7 cells (2×10^5^ cells/mL) were seeded into 96-well plates and treated with various concentrations of NOR (1, 3, 10, 30, 60 and 100 µM) in the presence or absence of RANKL (100 ng/mL) for 72 h.

For the MTT assay, 20 µL of MTT solution (5 mg/mL) was added and the cells were continuously incubated for an additional 4 h. Subsequently, the supernatants were removed, and the formazone crystals were dissolved using 150 µL of DMSO. The optical absorbance at 570 nm was read with a Model 1500 Multiskan spectrum microplate Reader (Thermo, Waltham, MA, USA).

For the LDH assay, the supernatants were collected, and the amounts of LDH released were measured using kits according to the manufacturer's instructions.

### Osteoclast differentiation

BMMs were seeded at a density of 2×10^5^ cells/mL in 48-well plates and treated with NOR (3, 10 and 30 µM) in the presence or absence of RANKL (100 ng/mL) and M-CSF (50 ng/mL) for 5 days. Osteoclasts were identified by TRAP staining according to the manufacturer's instructions. TRAP-positive multinuclear cells with greater than three nuclei were counted as osteoclasts.

RAW264.7 cells were seeded at a density of 2×10^5^ cells/mL in 48-well plates and treated with NOR (3, 10 and 30 µM) in the presence or absence of RANKL (100 ng/mL) for 5 days. The identification of osteoclasts is similar to those differentiated from BMMs.

### TRAP activity assay

RAW264.7 cells (2×10^5^ cells/mL) were seeded into 24-well plates and incubated with NOR (1, 3, 10, 30 and 60 µM), SB203580 (2 and 4 µM), U0126 (2 and 4 µM) or SP600125 (2 and 4 µM) in the presence or absence of RANKL (100 ng/mL) for 24 h. Cells were then lysed in 0.2% Triton X-100, and TRAP activity was detected using kits according to the manufacturer's instructions.

To identify which stage of osteoclast differentiation that NOR would exhibit most powerful interference effect, NOR (30 µM) was added into RAW264.7 cells and BMMs cultures that treated with RANKL (100 ng/mL) at 5 different time points. After exposure to NOR for 24 h, the culture media containing NOR was washed out and changed to NOR free culture media.The cells were continued to be stimulated with RANKL (100 ng/mL). Until day 5, cells were lysed in 0.2% Triton X-100, and TRAP activity was detected using kits according to the manufacturer's instructions.

### Bone resorption assay

The bone resorption function of osteoclasts derived from RAW264.7 cells induced by RANKL was analyzed using the bovine bone slice assay. Briefly, RAW264.7 cells (2×10^5^ cells/mL) were plated on slices and treated with NOR (3, 10 and 30 µM) in the presence or absence of RANKL (100 ng/mL) for 5 days. After this culture period, cells were removed completely. The slices were stained with toluidine blue. Resorption areas were observed under a light microscope and analyzed by Image-Pro Plus 6.0.

### Reverse-transcription polymerase chain reaction (RT-PCR) assay

The mRNA expressions of the osteoclastogenesis-related genes c-src, integrin αV, integrin β3, cathepsin K and MMP-9 in RAW264.7 cells, treated with NOR (3, 10 and 30 µM) for 24 h in the presence or absence of RANKL (100 ng/mL), were assessed using RT-PCR assay. Total RNA was extracted with TRIzol reagent according to the manufacturer's instructions. The concentration was determined by spectrophotometric optical density measurement at 260 nm and 280 nm. RNA (2 µg) was reverse-transcribed with M-MLV reverse transcriptase. A master mix containing the reaction buffer, dNTPs, Taq polymerase, and 2 µL of cDNA in a 25 µL reaction mixture was transferred to different PCR tubes, and β-actin served as a normalization control. The PCR products were determined using 1.2% agarose gel electrophoresis and Goldview staining. Images of the gels were analyzed using the Quantity One software (Bio-Rad, CA, USA) by comparing the relative density of objective straps and β-actin. Gene-specific primers used in RT-PCR were as follows: c-src forward 5′-TCCAGGCTGAGGAGTGGTACTTTGG-3′, reverse 5′-ATACGGTAGTGAGGCGGTGACACAG-3′; Integrin αV forward 5′-GTGTGTGGCCATTGCCTATCTC-3′, reverse 5′-GCATAGCCACAGGTTCATGGTTC-3′; Integrin β3 forward 5′-GCAGGTAGCTCTGTTCCCGTTC-3′, reverse 5′-AAGGCGCGTAAGCAATTTCAA-3′; MMP-9 forward 5′-CCTGTGTGTTCCCGTTCATCT-3′, reverse 5′-CGCTGGAATGATCTAAGCCCA-3′; cathepsin K forward 5′-GGAAGAAGACTCACCAGAAGC-3′, reverse 5′-GCTATATAGCCGCCTCCACAG-3′; β-actin forward 5′-AGCCATGTACGTAGCCATCC-3′, reverse 5′-CTCTCAGCAGTGGTGGTGAA-3′. The samples were pre-heated to 95°C and incubated for 35–40 PCR cycles. Each cycle consisted of a denaturation step at 95°C for 30 s, an annealing step at 60°C for 30 s, and an extension step at 72°C for 45 s.

### Gelatin zymography assay

MMP-9 activity was detected using the gelatin zymography assay. RAW264.7 cells (2×10^5^ cells/mL) were seeded into 24-well plates and treated with NOR (3, 10 and 30 µM) in the presence or absence of RANKL (100 ng/mL) for 24 h. The supernatants were collected and used as the samples. Cells were also collected, and proteins were extracted using lysis buffer (50 mM Tris-HCl, 150 mM NaCl, 0.02% NaN_3_, 1% NP-40) for 30 min. The protein concentration was determined using the Bradford assay. As the protein levels were not changed by RANKL or NOR treatments, the same volumes of these culture supernatants were used for the assays. The sample was electrophoresed on 8% SDS-polyacrylamide gels copolymerized with 1% gelatin. After electrophoresis, the gels were washed twice in 2.5% Triton X-100 and incubated in 1% Triton X-100, 5 mM CaCl_2_ and 5 µM ZnCl_2_ (pH 7.5) at 37°C for 36 h. The gels were stained with 0.1% Coomassie blue R250, and destained in 10% isopropanol and 10% acetic acid in H_2_O. MMP-9 was detected as transparent bands on the blue background of a Coomassie blue-stained gel.

### Western blotting assay

RAW264.7 cells (2×10^5^ cells/mL) were pre-treated with different concentrations of NOR (3, 10 and 30 µM) for 24 h and then stimulated with RANKL (100 ng/mL) for appropriate periods. Subsequently, the cells were washed twice with ice-cold PBS buffer (pH 7.2). Proteins were then extracted by using a protein extraction kit according to the manufacturer's instructions (Shangon, shanghai), and the protein concentration was determined using the Bradford assay. Samples were fractionated on a 10% SDS-PAGE gel and transferred to PVDF membranes. The membranes were blocked for 1 h at room temperature with 5% nonfat-milk and incubated with different antibodies. After being rinsed, the membranes were incubated with secondary antibodies for 1 h. The bands were visualized by using film exposure with ECL substrate.

### Immunoprecipitation assay

The accumulation of TRAF6-TAK1 complexes was detected using an immunoprecipitation assay which performed as following description. Briefly, RAW264.7 cells (2×10^5^ cells/mL) were pre-treated with various concentrations of NOR (3, 10 and 30 µM) for 24 h and then stimulated with RANKL (100 ng/mL) for 5 min. The cells were washed with ice-cold PBS and lysed in lysis buffer [25 mM HEPES (pH 7.7), 0.3 M NaCl, 1.5 mM MgCl_2_, 0.2 mM EDTA, 0.1% Triton X-100, 10 mM β-glycerophosphate, 1 mM Na_3_VO_4_, 1 mM PMSF, 1 mM dithiothreitol]. After centrifuged for 5 min at a speed of 12000 rpm, cell lysates (1 mL) containing 1.5 mg of total protein were incubated with 1 mg of TAK1 antibody and 20 mL of protein A/G-conjugated beads overnight. The beads were washed with lysis buffer for four times, and samples were centrifuged at 3000 g for 2 min and then resuspended in 20 mL of SDS-sample buffer (0.5 M Tris-HCl, pH 6.8, 20% glycerol, 2% SDS, 5% 2-mercaptoethanol, 4% bromophenol blue). For western blotting analysis, 10 mL of samples were used, and bands were visualized using film exposure with ECL substrate.

The ubiquitination of TRAF6 was also detected using an immunoprecipitation assay. The treatments of RAW264.7 cells and the extraction of protein were all performed as above description. The cell extracts were immunoprecipitated with antibody for TRAF6. The ubiquitination of TRAF6 was detected by immunoblotting with antibody. The bands were visualized using film exposure with ECL substrate.

### Electrophoretic mobility shift assay (EMSA)

The DNA-binding activity of NF-κB was detected with the electrophoretic mobility shift assay using a commercial kit (Pierce, USA). RAW264.7 cells (2×10^5^ cells/mL) were pre-treated with various concentrations of NOR (3, 10 and 30 µM) for 24 h and then stimulated with RANKL (100 ng/mL) for 30 min, and nuclear proteins were extracted. Biotin-labeled NF-κB-specific oligonucleotides were prepared as the labeled probe according to the manufacturer's instructions. Nuclear extracts were mixed with poly (dI-dC), labeled probe, binding buffer (100 mM NaCl, 30 mM HEPES, 1.5 mM MgCl_2_, 0.3 mM EDTA, 10% glycerol, 1 mM PMSF, 1 µg/µL aprotinin and 1 µg/µL leupeptin) and incubated at room temperature for 10 min. Then, 10 µL of protein-DNA complexes was subsequently fractionated on a gel electrophoresis at 10 V/cm for 1 h at 4°C with 6.5% polyacrylamide gels and transferred to a nylon membrane. The biotin end-labeled DNA was detected using a Streptavidin-HRP conjugate and a chemiluminescent substrate. The membrane was then exposed to X-ray film and finally analyzed using Quantity One software (Bio-Rad, CA, USA).

### Statistical analysis

Data were presented as the means±S.D. of three independent experiments. Statistical differences were assessed by one-way analysis of variance (ANOVA) followed by a post hoc Tukey's test. *P* values less than 0.05 (*P*<0.05) were accepted as a significant difference.

## References

[1] Ngian GS (2010) Rheumatoid arthritis. Aus Fam Physician 39: 626–628. 20877764

[2] Silman AJ, Pearson JE (2002) Epidemiology and genetics of rheumatoid arthritis. Arthritis Res 4 Suppl 3: 265–272. 10.1186/ar578 PMC3240153 12110146

[3] Karmakar S, Kay J, Gravallese EM (2010) Bone damage in rheumatoid arthritis: mechanistic insights and approaches to prevention. Rheum Dis Clin North Am 36: 385–404. 20510240 10.1016/j.rdc.2010.03.003 PMC2905601

[4] Pettit AR, Walsh NC, Manning C, Goldring SR, Gravallese EM (2006) RANKL protein is expressed at the pannus-bone interface at sites of articular bone erosion in rheumatoid arthritis. Rheumatology (Oxford) 45: 1068–1076. 16490750 10.1093/rheumatology/kel045

[5] Schett G (2007) Cells of the synovium in rheumatoid arthritis. Osteoclasts. Arthritis Res Ther 9: 203. 17316459 10.1186/ar2110 PMC1860063

[6] Lacey DL, Timms E, Tan HL, Kelley MJ, Dunstan CR, et al (1998) Osteoprotegerin ligand is a cytokine that regulates osteoclast differentiation and activation. Cell 93: 165–176. 9568710 10.1016/s0092-8674(00)81569-x

[7] Kong YY, Yoshida H, Sarosi I, Tan HL, Timms E, et al (1999) OPGL is a key regulator of osteoclastogenesis, lymphocyte development and lymph-node organogenesis. Nature 397: 315–323. 9950424 10.1038/16852

[8] Szekanecz Z, Koch AE (2007) Macrophages and their products in rheumatoid arthritis. Curr Opin Rheumatol 9: 289–295. 10.1097/BOR.0b013e32805e87ae 17414958

[9] Xu S, Wang Y, Lu J, Xu J (2011) Osteoprotegerin and RANKL in the pathogenesis of rheumatoid arthritis-induced osteoporosis. Rheumatol Int. [Epub ahead of print]. 10.1007/s00296-011-2175-5 22057136

[10] Berthelot JM, Le Goff B (2010) Rheumatoid arthritis and periodontal disease. Joint Bone Spine 77: 537–541. 20646949 10.1016/j.jbspin.2010.04.015

[11] Braun T, Zwerina J (2011) Positive regulators of osteoclastogenesis and bone resorption in rheumatoid arthritis. Arthritis Res Ther 13: 235. 21861862 10.1186/ar3380 PMC3239343

[12] Lewiecki EM (2009) Denosumab for joints and bones. Curr Rheumatol Rep 11: 196–201. 19604464 10.1007/s11926-009-0027-z

[13] Sharp JT, Tsuji W, Ory P, Harper-Barek C, Wang H, et al (2010) Denosumab prevents metacarpal shaft cortical bone loss in patients with erosive rheumatoid arthritis. Arthritis Care Res (Hoboken) 62: 537–544. 20391509 10.1002/acr.20172

[14] Li QL, Chou GX, Dou CG, Wang ZT, Huang F (1999) Compositions and anti-rheumatism effect of LEF fraction from the root of Lindera aggregate (Sims) Kosterm. J Plant Resour Environ 8: 1–6.

[15] Wang C, Dai Y, Yang J, Chou G, Wang C, et al (2007) Treatment with total alkaloids from Radix Linderae reduces inflammation and joint destruction in type II collagen-induced model for rheumatoid arthritis. J Ethnopharmacol 111: 322–328. 17204385 10.1016/j.jep.2006.11.031

[16] Luo Y, Liu M, Xia Y, Dai Y, Chou G, et al (2010) Therapeutic effect of norisoboldine, an alkaloid isolated from Radix Linderae, on collagen-induced arthritis in mice. Phytomedicine 17: 726–731. 20363113 10.1016/j.phymed.2010.01.013

[17] Wei ZF, Wang FY, Song J, Lu Q, Zhao P, et al (2012) Norisoboldine inhibits the production of interleukin-6 in fibroblast-like synoviocytes from adjuvant arthritis rats through PKC/MAPK/NF-κB-p65/CREB pathways. J Cell Biochem 113: 2785–2795. 22473817 10.1002/jcb.24156

[18] Cheng B, Li J, Du J, Lv X, Weng L, et al (2012) Ginsenoside Rb1 inhibits osteoclastogenesis by modulating NF-κB and MAPKs pathways. Food Chem Toxicol 50: 1610–1615. 22386813 10.1016/j.fct.2012.02.019

[19] Okamoto K, Takayanagi H (2011) Osteoclasts in arthritis and Th17 cell development. Int Immunopharmacol 11: 543–548. 21081190 10.1016/j.intimp.2010.11.010

[20] Broadhead ML, Clark JC, Dass CR, Choong PF, Myers DE (2011) Therapeutic targeting of osteoclast function and pathways. Expert Opin Ther Targets 15: 169–181. 21204734 10.1517/14728222.2011.546351

[21] Wilson TJ, Nannuru KC, Futakuchi M, Sadanandam A, Singh RK (2008) Cathepsin G enhances mammary tumor-induced osteolysis by generating soluble receptor activator of nuclear factor-kappaB ligand. Cancer Res 68: 5803–5811. 18632634 10.1158/0008-5472.CAN-07-5889

[22] Blair HC, Zaidi M (2006) Osteoclastic differentiation and function regulated by old and new pathways. Rev Endocr Metab Disord 7: 23–32. 17115268 10.1007/s11154-006-9010-4

[23] Blair JM, Zheng Y, Dunstan CR (2006) RANK ligand. Int J Biochem Cell Biol 39: 1077–1081. 17174136 10.1016/j.biocel.2006.11.008

[24] Logar DB, Komadina R, Prezelj J, Ostanek B, Trost Z, et al (2007) Expression of bone resorption genes in osteoarthritis and in osteoporosis. J Bone Miner Metab 25: 219–225. 17593491 10.1007/s00774-007-0753-0

[25] Schett G (2007) Erosive arthritis. Arthritis Res Ther 9 Suppl 1: S2. 10.1186/ar2166 PMC1924517 17634141

[26] Zou W, Kitaura H, Reeve J, Long F, Tybulewicz VL, et al (2007) Syk, c-Src, the alphavbeta3 integrin, and ITAM immunoreceptors, in concert, regulate osteoclastic bone resorption. J Cell Biol 12: 877–888. 10.1083/jcb.200611083 PMC2064061 17353363

[27] Arch RH, Gedrich RW, Thompson CB (1998) Tumor necrosis factor receptor-associated factors (TRAFs)-a family of adapter proteins that regulates life and death. Genes Dev 12: 2821–2830. 9744859 10.1101/gad.12.18.2821

[28] Mizukami J, Takaesu G, Akatsuka H, Sakurai H, Ninomiya-Tsuji J, et al (2002) Receptor activator of NF-kappaB ligand (RANKL) activates TAK1 mitogen-activated protein kinase kinase kinase through a signalling complex containing RANK, TAB2, and TRAF6. Mol Cell Biol 22: 992–1000. 11809792 10.1128/MCB.22.4.992-1000.2002 PMC134634

[29] Walsh MC, Kim GK, Maurizio PL, Molnar EE, Choi Y (2008) TRAF6 autoubiquitination-independent activation of the NFkappaB and MAPK pathways in response to IL-1 and RANKL. PLoS One 3: e4064. 19112497 10.1371/journal.pone.0004064 PMC2603309

[30] Landström M (2010) The TAK1-TRAF6 signalling pathway. Int J Biochem Cell Biol 42: 585–589. 20060931 10.1016/j.biocel.2009.12.023

[31] Krens SF, Spaink HP, Snaar-Jagalska BE (2006) Functions of the MAPK family in vertebrate-development. FEBS Letters 580: 4984–4990. 16949582 10.1016/j.febslet.2006.08.025

[32] Huang H, Chang EJ, Ryu J, Lee ZH, Lee Y, et al (2006) Induction of c-Fos and NFATc1 during RANKL-stimulated osteoclast differentiation is mediated by the p38 signaling pathway. Biochem Biophys Res Commun 351: 99–105. 17052691 10.1016/j.bbrc.2006.10.011

[33] Ikeda F, Matsubara T, Tsurukai T, Hata K, Nishimura R, et al (2008) JNK/c-Jun signaling mediates an anti-apoptotic effect of RANKL in osteoclasts. J Bone Miner Res 23: 907–914. 18251700 10.1359/jbmr.080211

[34] Lee MS, Kim HS, Yeon JT, Choi SW, Chun CH, et al (2009) GM-CSF regulates fusion of mononuclear osteoclasts into bone-resorbing osteoclasts by activating the Ras/ERK pathway. J Immunol 183: 3390–3399. 19641137 10.4049/jimmunol.0804314

[35] Karin M, Yamamoto Y, Wang QM (2004) The IKK NF-κB system: a treasure trove for drug development. Nat Rev Drug Discov 3: 17–26. 14708018 10.1038/nrd1279

[36] Novack DV (2011) Role of NF-κB in the skeleton. Cell Res 21: 169–1682. 21079651 10.1038/cr.2010.159 PMC3193402

[37] Ishii J, Kitazawa R, Mori K, McHugh KP, Morii E, et al (2008) Lipopolysaccharide suppresses RANK gene expression in macrophages by down-regulating PU.1 and MITF. J Cell Biochem 105: 896–904. 18759249 10.1002/jcb.21886

[38] Zhao Q, Wang X, Liu Y, He A, Jia R (2010) NFATc1: functions in osteoclasts. Int J Biochem Cell Biol 42: 576–579. 20035895 10.1016/j.biocel.2009.12.018

[39] Sharma SM, Bronisz A, Hu R, Patel K, Mansky KC, et al (2007) MITF and PU.1 recruit p38 MAPK and NFATc1 to target genes during osteoclast differentiation. J Biol Chem 282: 15921–1599. 17403683 10.1074/jbc.M609723200

[40] Chou GX, Norio N, Ma CM, Wang ZT, Masao H (2005) Isoquinoline alkaloids from Lindera aggregata. Chin J Nat Med 5: 272–275.

[41] Distler JH, Huber LC, Hueber AJ, Reich CF, 3rd Gay S, et al (2005) The release of microparticles by apoptotic cells and their effects on macrophages. Apoptosis 10: 731–741. 16133865 10.1007/s10495-005-2941-5

